# Construction and Analysis of the Model of Energy Metabolism in *E. coli*

**DOI:** 10.1371/journal.pone.0055137

**Published:** 2013-01-30

**Authors:** Zixiang Xu, Xiao Sun, Jibin Sun

**Affiliations:** 1 Key Laboratory of Systems Microbial Biotechnology, Tianjin Institute of Industrial Biotechnology, Chinese Academy of Sciences, Tianjin, China; 2 State Key Laboratory of Bioelectronics, Southeast University, Nanjing, China; Virginia Commonwealth University, United States of America

## Abstract

Genome-scale models of metabolism have only been analyzed with the constraint-based modelling philosophy and there have been several genome-scale gene-protein-reaction models. But research on the modelling for energy metabolism of organisms just began in recent years and research on metabolic weighted complex network are rare in literature. We have made three research based on the complete model of *E. coli*’s energy metabolism. We first constructed a metabolic weighted network using the rates of free energy consumption within metabolic reactions as the weights. We then analyzed some structural characters of the metabolic weighted network that we constructed. We found that the distribution of the weight values was uneven, that most of the weight values were zero while reactions with abstract large weight values were rare and that the relationship between *w* (weight values) and *v* (flux values) was not of linear correlation. At last, we have done some research on the equilibrium of free energy for the energy metabolism system of *E. coli*. We found that 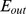 (free energy rate input from the environment) can meet the demand of 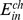 (free energy rate dissipated by chemical process) and that chemical process plays a great role in the dissipation of free energy in cells. By these research and to a certain extend, we can understand more about the energy metabolism of *E. coli*.

## Introduction

Since various ‘Omics’ datasets are becoming available, biology has transited from a data-poor to a data-rich environment. Systems biology has become a rapidly growing field as well [1]. Genome-scale models of metabolism have only been analyzed with the constraint-based modelling philosophy [2], [3]. Genome-scale network models of diverse cellular processes have been generated and there have been several genome-scale GPR (gene-protein-reaction) models [4]–[10]. An extensive set of methods for analyzing these genome-scale models have also been developed and have also been applied to study a growing number of biological problems [12], [13]. But research on the energy metabolism of organisms just begin in recent years, such as FVA (flux variability analysis) [14], [15] and EBA (energy balance analysis) [16]–[18], and so on. All these methods are depended on the modelling of energy metabolism system of organisms. Data of Gibbs free energy of formation of every compound and Gibbs free energy change of every reaction is the core in this kind of modelling. Up to now, the most detailed genome-scale GPR model is the iAF1260 version of *E. coli* [5], but the modelling of it’s energy metabolism is still incomplete [5]. There are 2381 reactions (not including the reaction defined for growth) and 1039 metabolites in *E. coli*_iAF1260, and apart from 304 EX_ & DM_ reactions (The text ‘EX_’ denotes an exchange reaction for a metabolite that can enter or leave the extra-cellular compartment. ‘DM_’ reactions are similar and signify compounds that the degradation pathway is unknown), the reconstructed reaction number is 2077 [5]. By the newest group contribution method (GCM), 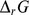 of 1996 reactions (96%) and 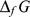
of 872 compounds (84%) can be estimated [19]–[22]. There leaves Gibbs free energy change (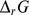) of 81 reactions (4%) and Gibbs free energy of formation (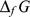) of 167 compounds (16%) unknown for *E. coli*_iAF1260 [5], [19]. We have complemented, by computational method, the remaining unknown 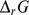 (including the standard Gibbs free energy change 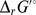 and the free energy change of reaction at 1 mM concentrations for all species 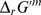) and 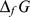 (just the standard Gibbs free energy formation 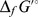). Energy metabolism models of other organisms, as we know, do not exist up to now. Research on metabolic weighted complex network are also rare in literature except that Almaas has used flux value as the weight of metabolic network [23].

In this paper, we have done three research on the complete model of *E. coli*’s energy metabolism. First, we construct a metabolic weighted network using the rates of free energy consumption in metabolic reactions as the network weights. Then we did some research on some structural characters of the metabolic weighted network we constructed. At last, we did some research on the equilibrium of free energy for the energy metabolism system of *E. coli*.

## Materials and Methods

Before constructing the metabolic weighted network, we complement the remained unknown 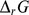 and 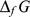 in *E. coli* _iAF1260. Then we construct the model using the rates of free energy consumption in metabolic reactions as the network weights. At first, we draw the metabolic unweighted network of *E. coli*; we then calculate the flux distribution of *E. coli*_iAF1260; the third, we calculate the weights of the metabolic weighted network of *E. coli* that we will construct; at last, we calculated the input and output of free energy about *E. coli*.

### Complement the Remained Unknown Free Energy Change of *E. Coli*_iAF1260

#### 1) Infer the unknown standard Gibbs free energy of formation for 167 compounds

The stoichiometric matrix, ***S***, is the center-piece of a mathematical representation of genome-scale metabolic networks. It represents each reaction as a column and each metabolite as a row, where each numerical element is the corresponding stoichiometric coefficient. In the calculation of 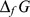 of compounds or 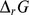 of reactions, we should use “reaction with marvin charges (pH7)” as the stoichiometry [5].

There are 1039 metabolites in the iAF1260 model, and if distinguishing the different compartments in the cell, i.e. [c] (cytoplasm), [e] (extracellular space), and [p] (periplasm), the total number of metabolites in the model is 1668. So there are 1668 rows in the stoichiometric matrix ***S***. For a certain compound even in different compartments, the free energy change of formation is the same. In these 1039 metabolites, Gibbs free energy of formation of 872 compounds (84%) can be estimated by group contribution method [19]–[22], while 167 compounds (16%) remained unknown for *E. coli*_iAF1260. There are 2381 reactions in the iAF1260 model, so there are 2381 columns in the stoichiometric matrix ***S***. For those 2077 reconstructed reactions, Gibbs free energy change of 1996 reactions (96%) can be estimated by group contribution method [19]–[22], while 81 reactions (4%) remained unknown for *E. coli*_iAF1260.

From the equation (1) and (2) of the paper [19], we can infer that
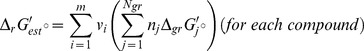
(1)

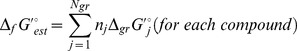
(2)

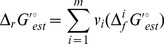
(3)


Where 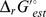 is the estimated 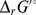; 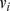 is the stoichiometric coefficient of species *i* in the reaction, and *m* is the number of species involved in the reaction; 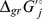 is the contribution of group *j*; *n*_j_ is the number of instances of group *j* in the molecular structure; *N*_gr_ is the number of groups for which 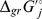 is known; 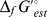 is the estimated 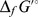; 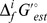 is the estimated Gibbs free energy of formation of *i*-th species. Equation (3) reflects the relationship between 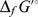 and 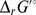
for a reaction.

From those 167 compounds with unknown 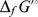, we seek out their involved reactions. Gibbs free energy change of one reaction can not be calculated, if a compound with unknown free energy change of formation appears in it. But the case is not for all. In the reactions involving the structural group with unknown energy, the structural group appears on both sides of the reaction, which means it cancels out of “group energy change” for the reaction. That is to say, while the compounds contain a structural group with unknown energy (such as “R” group, a pseudoatom) and appear in a reaction and the reaction does involve a change in the group, we can still calculate and estimate the energy change of the reaction, but we cannot estimate the formation energies of the compounds. Because a reaction with unknown 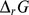 may include several compounds with unknown 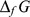, from equation (3), the values of those 81 unknown 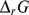 and the values of those 167 unknown 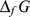 may be interdependent. So we cannot infer the unknown 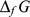 of compounds in a reaction just from the value of known 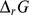 of this reaction, but we may infer the 167 unknown 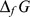 of compounds from all of the values of known 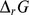 by solving their simultaneous equations.

Now we infer these unknown 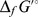 from the known 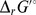 data of those involved reactions. We use vector ***X*** (with dimension 167×1) to indicate the values of those unknown 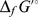 of 167 compounds; Use vector ***P*** (with dimension 1668×1) to indicate the values of 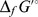 of entire 1668 compounds in the model of *E. coli*_iAF1260; Let the value of ***P***(i), *i*-th sub-variable of ***P*** **,** be 0, if the 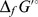 of *i*-th compound is unknown; Use vector ***F*** (with dimension 2381×1) to indicate the values of 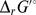 of entire 2381 reactions in the model of *E. coli*_iAF1260. From equation (3), we can obtain the following equation
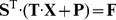
(4)


Where ***T*** (with dimension 1668×167) is the transfer matrix from vector ***X*** to the vector indicating the values of 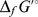 of entire 1668 compounds in the model of *E. coli*_iAF1260; ***S*** (with dimension 1668×2381) is the stochiomatrix of *E. coli*_iAF1260, *S* ^T^ is its transpose. Further we obtain
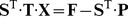
(5)


Write it as
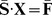
(6)


Where 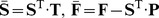


The dimension of vector 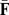 is 2381, and there are 1996 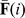 with known 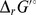. But there are 244 rows of 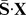 with unknown 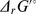 in the corresponding 1996 rows of 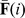, for the product of the corresponding rows of 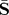 and ***X*** includes the sub-variables of ***X*** and while the remained 1752 rows of 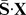 do not include the sub-variables of ***X***. So we can use these 244 rows from 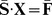 to get a new equation
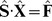
(7)


Where the dimensions of matrix 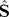 and 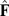 are respectively 244×167 and 244×1. By solving equation (7), we can obtain the 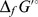 values of those 167 compounds which are unknown previously. Although equation (7) is not an exact equation (the row rank of matrix 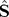 is not equal to the column rank of matrix 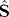), its solution is of least-squares.

#### 2) Calculate the unknown Gibbs free energy change of 81 reactions

Conversely, we now use the obtained 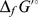 data to calculate the 81 unknown 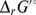. The method is to substitute the solution value (defined as **X**_0_) of 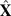 which we got from equation (7) to the equation (4), and by a simple calculation, we got the vector ***F***_0_ of 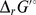


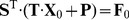
(4′)


Now all the sub-variables of ***F***_0_ are known, so we can now obtain the 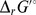 values of those 81 reactions which are unknown previously.

#### 3) Adjust 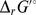
(the standard Gibbs free energy change) to 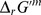 (the free energy change of reaction at 1mM concentrations)

The 1M reference state for the metabolite concentrations on which 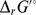 is based does not accurately reflect the metabolite concentrations found in the cell (approximately 1 mM). Thus, we should computationally adjust all estimated 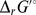 to the free energy change of reaction at 1 mM concentrations for all species, 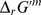. The relationship between 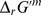 and 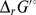
is as follows [5], [19].
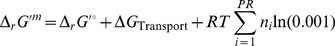
(8)


Where *R* is the universal gas constant; *T* is the temperature assumed to be 298 K; *n*_i_ is the stoichiometric coefficient of compound *i* in the reaction (*n*_i_ is negative for reactants and positive for products); *PR* is the set of products and reactants in this reaction. Note also that for H_2_, we should substitute 0.000034 for 0.001; For O_2_, we should substitute 0.000055 for 0.001; For H_2_O and H^+^, we should not include these compounds in the concentration portion of the calculation at all [5], [19]. Here, all of the 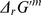
values reported in our work have included the energy contribution of the transmembrane electrochemical potential and proton gradient for all reactions involving transport across the cytoplasmic membrane.

### Unweighted Network of *E. Coli*_iAF1260

The general features of *E. coli*_iAF1260 are given in Ref. [5]. Two SBML (systems biology markup language) format files to the model *E. coli*_iAF1260 can be downloaded from the supplementary information of Ref. [5]. The *in silico* model that we used is *E. coli*_iAF1260_flux1.xml. SBML file properties are also given in Ref. [5]. The dimensions of **rxns**, **mets**, and **genes** are respectively 2382, 1668, 1261. The minimal media of *in silico* model is also an important aspect. The computational minimal media of *E. coli*_iAF1260 is also included in the supplementary information of Ref. [5]. In the method of constraint-based analysis, the biomass objective function (BOF) should be defined. The BOF was generated by defining all of the major and essential constituents that make up the cellular biomass content of *E. coli* [5]. Gene-protein-reaction associations embodied in **rxnGeneMat** matrix, which is a matrix with as many rows as there are reactions in the model and as many columns as there are genes in the model. The *i*th row and *j*th column contains a one if the *j*th gene in **genes** is associated with the *i*th reaction in **rxns** and zero otherwise. The simulation condition (the nutrients and the uptake rates of the nutrients) of this paper is the same as in the file.

### Flux Distribution of *E. Coli*_iAF1260

We now calculate the flux distribution of *E. coli*_iAF1260. The computational method we use is flux balance analysis (FBA) [11], one of the fundamental genome-scale phenotypic calculations, which can simulate cellular growth. FBA is based on linear optimization of an objective function, which typically is biomass formation. Given an uptake rate for key nutrients and the biomass composition of the cell (usually in mmol component gDW^−1^ and defined in the biomass objective function), the maximum possible growth rate of the cells can be predicted *in silico*.
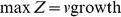
(9)


Subject to
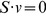
(10)

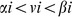
(11)


**Figure 1:**
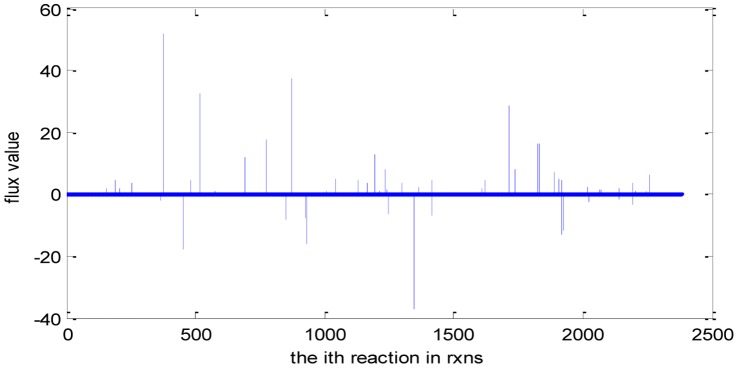
Flux distribution of *E. coli*_iAF1260. *X*-axis indicating every reaction in **rxns** (the order is as the same as in **rxns**, total 2382) and *y*-axis indicating the value of its corresponding flux (unit is mmol gDW^−1^h^−1^). **Rxns** is the reaction set in the model.

Where ***S*** is the stoichiometric matrix, and *α_i_* and *β_i_* define the bounds through each reaction *v_i_*. The flux range was set arbitrarily high for all internal reactions so that no internal reaction restricted the network, with the exception of irreversible reactions, which have a minimum flux of zero. The inputs to the system were restricted to a minimal media. We use the COBRA toolbox [11] to carry out this computation of FBA. The flux distribution of *E. coli*_iAF1260 is illustrated in **Fig. 1**.

### Metabolic Weighted Network Construction for *E. Coli*_iAF1260

By the newest group contribution method, 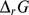
of 1996 reactions (96%) and 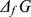 of 872 compounds (84%) can be estimated [19]–[22]. We have complemented, by computation method, the remained unknown 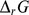 (including the standard Gibbs free energy change 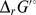 and the free energy change of reaction at 1 mM concentrations for all species 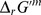) and 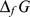 (just the standard Gibbs free energy formation 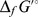). So a complete set of the data of free energy changes for reactions in *E. coli* can be obtained. .

Now we can construct a metabolic weighted network. There is not a standard manner of determining reaction edge weights and Almaas has used flux value as the weight of metabolic network [23]. Here we use the rates of free energy dissipation in metabolic reactions as the network weights. For 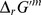 of a reaction is the free energy dissipation in unit mol while flux is the passed mol number in unit time (as second). So the rate of free energy dissipation in every reaction is the multiplying product of the flux in this reaction and the free energy change of this reaction.
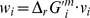
(12)


Where 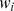 is the weight of *i*-th edge (i.e. reaction) of metabolic network, 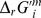 is the free energy change of *i*-th reaction and 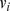 is the flux value of *i*-th reaction.

### Calculation of Input and Output of Free Energy in *E. Coli*_iAF1260

For an open system at nonequilibrium steady state, from the theory of system science, its free energy rate dissipated by the system, 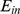, is in absolute value equal to the free energy rate input by the environment, 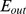.
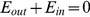
(13)


We also distinguish the free energy rate 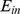 to 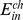 and 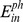 respectively dissipated by chemical process and by physical process that take place in the cell (eq. 14a), while the free energy rate input from environment and through physical process can be neglected(eq. 14b).
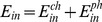
(14a)

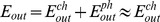
(14b)


For *E. coli*, we have known all of its reactions, the values of corresponding 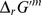 and the values of corresponding flux, so we can calculate its 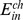 and 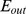 using the following equation (15) and (16).
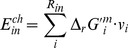
(15)


Where 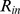 is the set of reactions of the metabolic network excluding EX_ & DM_ reactions (The text ‘EX_’ denotes an exchange reaction for a metabolite that can enter or leave the extra-cellular compartment. ‘DM_’ reactions are similar and signify compounds that the degradation pathway is unknown), 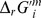 is the free energy change of *i*-th reaction in 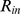 and 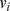 is the flux value of *i*-th reaction in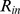.
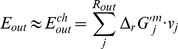
(16)


Where 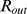 is the set of EX_ & DM_ reactions of the metabolic network, 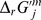 is the free energy change of *j*-the reaction in 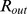 and 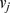 is the flux value of *j*-th reaction in 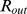.

## Results and Discussion

### Complement the Remained Unknown Free Energy Change of *E. Coli*_iAF1260

With our method, we obtain Gibbs free energy change (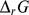) of 81 reactions (see **Table S1**) and Gibbs free energy of formation (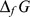) of 167 compounds (see **Table S2**) which are previously unknown for *E. coli*_iAF1260. We add our computed 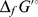 of those 167 compounds to the former known 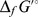 of 872 compounds, and obtain a complete set of 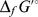 of *E. coli*_iAF1260. The entire 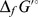 values of *E. coli*_iAF1260 are consistent with the known 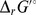 of 1996 reactions (see **Table S2** and **Table 1** **)**. So we conclude that our computed 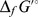 of those 167 compounds can also agree with the unknown 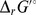 of 81 reactions. Up to now, there is no experimental data in literatures to test the 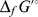 values of those 167 compounds and the 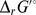 values of those 81 reactions.

**Table 1:** Our computation result comparing with Ref. [5].

	A	B	C	D
A	81	244	1752	1996
B	can’t compare	consistent	consistent	consistent

Note: Row A – Number in *E. coli*_iAF1260; Row B – Our computation result comparing with Ref. [5].

Column A – reactions with unknown 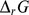; .

Column B – reactions with known 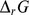 but involving compounds with unknown 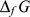; .

Column C – reactions with known 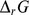 but not involving compounds with unknown 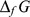; .

Column D – total reactions with known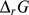.

It is important to know free energies for all metabolites and reactions in *E. coli* by using our method. First of all, the reason why GCM can’t calculate the free energies for all metabolites and reactions in *E. coli* or other organisms is that the free energies of some molecular substructures are present in organic-inorganic complexes involving iron, nickel, or cobalt for which the new group contribution method has not been designed [19]. So if we use large scale free energy datasets, not confined to *E. coli*, such as free energies for reaction of KEGG [19], we will get free energies for more metabolites which can’t be calculated by the GCM in ref. [19]. Even more, we can estimate some of the free energies of some molecular substructures in organic-inorganic complexes. So the method in our paper will directly contribute to GCM. At the same time, free energies for reactions are useful reference in determine the directions of reactions in cell [5], [17] and can also be used as constraints in FBA [18], so if we know all the free energies of reactions for an organism, we can better carry out these tasks.

### Some Structural Characters of the Metabolic Weighted Network of *E. Coli*_iAF1260

#### 1) Uneven distribution of the weight values of the metabolic weighted network of *E. coli*_iAF1260

**Figure 2:**
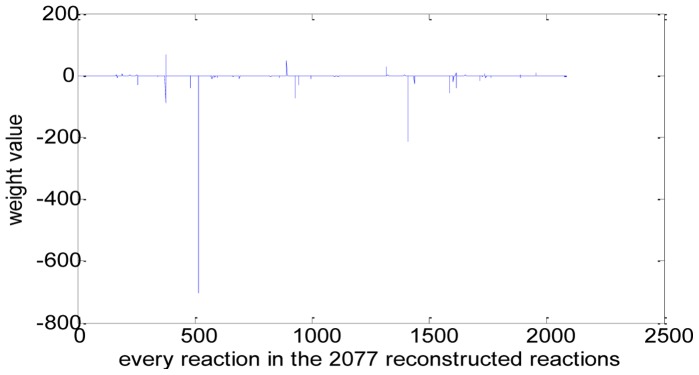
Weight value distribution of the metabolic network of *E. coli*_iAF1260. *X*-axis indicates every reaction in the reconstructed reactions (the order is as the same as in **rxns**, total 2077) and *y*-axis indicates the value of its corresponding weight. **rxns** is the reaction set in the model.

We can calculate the weight values of the metabolic network of *E. coli*_iAF1260 using the above equation (12) (see **Table S2**). We can easily find that the distribution of the weight values is uneven and that most of the weight values are near zero while reactions with abstract large weight values are rare, illustrating in **Fig. 2** and **Table 2**. The reason for the uneven distribution of weight values maybe lies in the uneven distribution of fluxes and the uneven distribution of Gibbs free energy change of reactions. From the uneven distribution of weight values, we can learn that there just are some main channels of free energy dissipation in the physiological process of *E. coli*. **Table 3** has illustrated some reaction channels which have large weight values and Table 4 gives the functions of these reactions.

**Table 2:** *w* scopes, number of reactions (NR) and their percentages.

*w* scopes	<−200	−200∼−100	−100∼−50	−50∼0	0	0∼40	40∼100	>100
NR	2	0	3	248	1753	69	2	0
%	0.1	0	0.14	11.94	84.40	3.32	0.1	0

**Table 3:** *w* scopes, number of reactions (NR) and reaction names (RM).

*w* scopes	<−200	−100∼−50	>40
NR	2	3	2
RM	CYTBO3_4ppNADH16pp	ATPM GLCptsppPDH	ATPS4rppGAPD

**Table 4:** Reaction names (RM) and their corresponding related genes.

RM	ATPM	ATPS4rpp	CYTBO3_4pp	GLCptspp	GAPD	NADH16pp	PDH
Equations	[c] : atp+h2o –> adp+h+pi	adp[c]+(4) h[p]+pi[c]< = = >atp[c]+(3) h[c]+h2o[c]	(4) h[c]+(0.5) o2[c]+q8h2[c] –> (4) h[p]+h2o[c]+q8[c]	[c] : bglycogen+pi –> g1p	[c] : g3p+nad+pi < = = >13dpg+h+nadh	(4) h[c]+nadh[c]+q8[c] –> (3) h[p]+nad[c]+q8h2[c]	[c] : coa+nad+pyr –> accoa+co2+ nadh
genes		b3736, b3737b3738, b3739b3731,b3732b3733,b3734b3735	b0429, b0430b0431, b0432	b2415, b2416b2417, b2418b2419b1621,b1101b1817,b1818b1818	b1779	b2276, b2277b2278, b2279b2280, b2281b2282, b2283b2284, b2285b2286, b2287b2288	b0114, b0115b0116

#### 2) Reactions of large weight values and their related genes

**Table 3** shows high *w* scopes, corresponding reaction number within these scopes and these reaction names, and we call these reactions the highly-dissipative reactions in the energy metabolism of *E. coli*. We examined into these large weights and found that they were the result of joint action from flux and free energy dissipation, while their 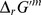 values were not the highest level. **Table 4** gives all of the genes related to these reactions and the rules among genes in these reactions (rules are defined as the relationship among genes catalyzing a reaction such as “AND, OR, NOT” and these rules can be found in Supplementary Information 1 of [5]), and we find that all of them are not essential genes from the literature [5]. This is important in the energy metabolism of *E. coli*, the deletion or loss of one gene will not result in death, and this may be due to the result of evolution.

#### 3) Correlation between the weight values and the flux values

**Figure 3:**
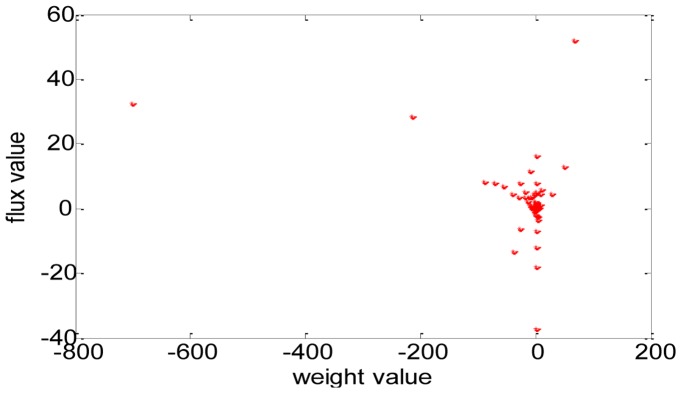
The scatter diagram (*w*, *v*). *X*-axis indicates *w* and *y*-axis indicates *v*.

By comparing the distribution of the weight values (**Fig. 2**) with the distribution of fluxes (**Fig. 1**), we can also find that they are not consistent. **Fig. 3** is the scatter diagram (*w*, *v*), 2077 data pairs in total. Many data pairs are superposition and locate at the same place. From the diagram, we can easily find that the relationship between *w* and *v* is not of linear correlation. So we can’t say that a reaction with high flux has a corresponding high weight and vice verse, and in other words, we can’t say that a reaction with high flux will dissipate more free energy. In fact, many different flux values correspond to the same weight value.

Although there is no consistency between flux values and energetic weights, energetic weights are very useful and important in determine the distribution of reaction fluxes. We have defined energetic weights as the rates of free energy dissipations, i.e. the multiplying product of the reaction fluxes and the free energy changes of reactions. Free energy dissipation can be regarded as the counterpart of entropy increase. Based on maximum entropy production principle (MEPP), the authors of the paper have developed a method to improve the prediction accuracy of flux balance analysis [24].

### Equilibrium of Free Energy in the Energy Metabolism of *E. Coli*_iAF1260

There are 2077 reactions in 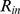 and 304 reactions in 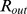 of *E. coli*_iAF1260. The values of 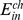 and 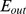 which we calculated are respectively −1424.7 and 1890.1, see **Table 5**. So 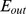 can meet the demand of 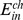 and 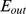 is a little more than 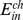. The absolute difference between 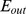 and 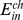 is an estimation of 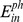, and we can find that it takes about a quarter of 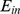, so we can conclude that chemical process plays a great role in the dissipation of free energy in cells while physical process can not be ignored.

**Table 5:** Equilibrium of free energy.

	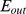	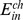	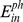
value	1890.1	−1424.7	−465.4
%	100%	75.37%	24.63%

### Conclusions

In this paper, we constructed a metabolic weighted network by using the rates of free energy consumption within metabolic reactions as the network weights. We found several important and interesting results: 1) the distribution of the weight values was uneven; 2) the relationship between w (weight values) and v (flux values) was not of linear correlation; 3) by analyzing of the free-energy equilibrium for the energy metabolism system of *E. coli*, we found that it is chemical process other than physical process that plays a great role in the dissipation of free energy in cells. By these research and to a certain extend, we can understand more about the energy metabolism of *E. coli*.

In our next step, we will conduct a similar type of analysis for different organisms using some of the other readily available constraint-based models and run the baseline simulations for growth in different carbon substrate environments. We will also do FBA analysis including energetic weighting as an additional constraint to bias flux distributions.

## Supporting Information

Table S1Standard Gibbs free energy formation 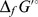
of 167 compounds.(XLS)

Table S2Gibbs free energy change (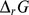) of 81 reactions (including the standard Gibbs free energy change 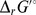 and the free energy change of reaction at 1 mM concentrations for all species 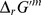), flux distribution and weight values.(XLS)

## References

[1] Bork P (2005) Is there biological research beyond Systems Biology? A comparative analysis of terms. Molecular Systems Biology.1: Art. No.2005.0012. 10.1038/msb4100016 PMC1681443 16729047

[2] Price ND, Papin JA, Schilling CH, Palsson BO (2003) Genome-scale microbial *in silico* models: the constraints-based approach. Trends in Biotechnology. 21(4): 162–169. 10.1016/S0167-7799(03)00030-1 12679064

[3] Price ND, Reed JL, Palsson BO (2004) Genome-scale models of microbial cells: evaluating the consequences of constraints. Nature Reviews Microbiology. 2(11): 886–897. 10.1038/nrmicro1023 15494745

[4] Reed JL, Vo TD, Schilling CH, Palsson BO (2003) An expanded genomescale model of *Escherichia coli* K-12 (iJR904 GSM/GPR). Genome Biology. 4: R54. 10.1186/gb-2003-4-9-r54 PMC193654 12952533

[5] Feist AM, Henry CS, Reed JL, Krummenacker M, Joyce AR, et al. (2007) A genome-scale metabolic reconstruction for *Escherichia coli* K-12 MG1655 that accounts for 1260 ORFs and thermodynamic information. Molecular Systems Biology. 3: Art. No.121. 10.1038/msb4100155 PMC1911197 17593909

[6] Scott AB, Bernhard ØP (2005) Genome-scale reconstruction of the metabolic network in *Staphylococcus aureus* N315: an initial draft to the two-dimensional annotation. BMC Microbiology. 5: Art. No.8. 10.1186/1471-2180-5-8 PMC1079855 15752426

[7] Thiele I, Vo TD, Price ND, Palsson BØ (2005) Expanded metabolic reconstruction of *Helicobacter pylori* (iIT341 GSM/GPR): an *in silico* genome-scale characterization of single- and double-deletion mutants. Journal of Bacteriology. 187 (16) 5818–5830. 10.1128/JB.187.16.5818-5830.2005 PMC1196094 16077130

[8] Feist AM, Scholten JC, Palsson BØ, Brockman FJ, Ideker T (2006) Modeling methanogenesis with a genome-scale metabolic reconstruction of Methanosarcina barkeri. Molecular Systems Biology. 2: Art. No.2006.0004. 10.1038/msb4100046 PMC1681478 16738551

[9] Duarte NC, Herrgård MJ, Palsson BØ (2004) Reconstruction and validation of *Saccharomyces cerevisiae* iND750, a fully compartmentalized genome-scale meta-bolic model. Genome Research. 14(7): 1298–1309. 10.1101/gr.2250904 PMC442145 15197165

[10] Oh YK, Palsson BO, Park SM, Schilling CH, Mahadevan R (2007) Genome-scale reconstruction of metabolic network in *Bacillus subtilis* based on high-throughput phenotyping and gene essen-tiality data. The Journal of Biological Chemistry. 282 (39): 28791–28799. 10.1074/jbc.M703759200 17573341

[11] Becker SA, Feist AM, Mo ML, Hannum G, Palsson BO, et al (2007) Quantitative prediction of cellular metabolism with constraint-based models: the COBRA Toolbox. Nature Protocols. 2(3): 727–738. 10.1038/nprot.2007.99 17406635

[12] Feist AM, Palsson BØ (2008) The growing scope of applications of genome-scale metabolic reconstructions using Escherichia coli. Nature Biotechnology. 26(6): 659–667. 10.1038/nbt1401 PMC3108568 18536691

[13] Di Ventura B, Lemerle C, Michalodimitrakis K, Serrano L (2006) From *in vivo* to *in silico* biology and back. Nature. 443 (7111): 527–533. 10.1038/nature05127 17024084

[14] Mahadevan R, Schilling CH (2003) The effects of alternate optimal solutions in constraint-based genome-scale metabolic models. Metabolic Engineering. 5: 264–276. 10.1016/j.ymben.2003.09.002 14642354

[15] Segura D, Mahadevan R, Juárez K, Lovley DR (2008) Computational and Experimental Analysis of Redundancy in the Central Metabolism of *Geobacter sulfurreducens*. PLoS Computational Biology. 4(2): e36. 10.1371/journal.pcbi.0040036 PMC2233667 18266464

[16] Beard DA, Liang SD, Qian H (2002) Energy Balance for Analysis of Complex Metabolic Networks. Biophysical Journal. 83: 79–86. 10.1016/S0006-3495(02)75150-3 PMC1302128 12080101

[17] Yang F, Qian H, Beard DA (2005) Ab initio prediction of thermodynamically feasible reaction directions from biochemical network stoichiometry. Metabolic Engineering. 7: 251–259. 10.1016/j.ymben.2005.03.002 16140239

[18] Beard DA, Babson E, Curtis E, Qian H (2004) Thermodynamic constraints for biochemical networks. Journal of Theoretical Biology. 228: 327–333. 10.1016/j.jtbi.2004.01.008 15135031

[19] Jankowski MD, Henry CS, Broadbelt LJ, Hatzimanikatis V (2008) Group Contribution Method for Thermo-dynamic Analysis of Complex Metabolic Networks. Biophysical Journal. 95: 1487–1499. 10.1529/biophysj.107.124784 PMC2479599 18645197

[20] Henry CS, Jankowski MD, Broadbelt LJ, Hatzimanikatis V (2006) Genome-Scale Thermodynamic Analysis of *Escherichia coli* Metabolism. Biophysical Journal. 90: 1453–1461. 10.1529/biophysj.105.071720 PMC1367295 16299075

[21] Henry CS, Broadbelt LJ, Hatzimanikatis V (2007) Thermodynamics-Based Metabolic Flux Analysis. Biophysical Journal. 92: 1792–1805. 10.1529/biophysj.106.093138 PMC1796839 17172310

[22] Hatzimanikatis V, Li CH, Ionita JA, Henry CS, Jankowski MD, et al (2005) Exploring the diversity of complex metabolic networks. Bioinformatics. 21(8): 1603–1609. 10.1093/bioinformatics/bti213 15613400

[23] Almaas E, Kovács B, Vicsek T, Oltvai ZN, Barabási AL (2004) Global organization of metabolic fluxes in the bacterium *Escherichia coli*. Nature. 427(6997): 839–843. 10.1038/nature02289 14985762

[24] Zhu Y, Song J, Xu Z, Sun J, Zhang Y, Li Y, Ma Y (2012) Development of thermodynamic optimum searching (TOS) to improve the prediction accuracy of flux balance analysis. Biotechnology and Bioengineering. published online: DOI: 10.1002/bit.24739. 10.1002/bit.24739 23042478

